# Optimal seasonal schedule for the production of isoprene, a highly volatile biogenic VOC

**DOI:** 10.1038/s41598-024-62975-3

**Published:** 2024-05-29

**Authors:** Yoh Iwasa, Rena Hayashi, Akiko Satake

**Affiliations:** Department of Biology, Faculty of Science, Kyushu University, 744 Motooka, Nishi-Ku, Fukuoka, 819-0395 Japan

**Keywords:** Volatility, Marginal value of leaf area, Pontryagin’s maximum principle, Post-risk enhancement, Immediate impact, The impact of future expectations, Evolutionary ecology, Applied mathematics

## Abstract

The leaves of many trees emit volatile organic compounds (abbreviated as BVOCs), which protect them from various damages, such as herbivory, pathogens, and heat stress. For example, isoprene is highly volatile and is known to enhance the resistance to heat stress. In this study, we analyze the optimal seasonal schedule for producing isoprene in leaves to mitigate damage. We assume that photosynthetic rate, heat stress, and the stress-suppressing effect of isoprene may vary throughout the season. We seek the seasonal schedule of isoprene production that maximizes the total net photosynthesis using Pontryagin’s maximum principle. The isoprene production rate is determined by the changing balance between the cost and benefit of enhanced leaf protection over time. If heat stress peaks in midsummer, isoprene production can reach its highest levels during the summer. However, if a large portion of leaves is lost due to heat stress in a short period, the optimal schedule involves peaking isoprene production after the peak of heat stress. Both high photosynthetic rate and high isoprene volatility in midsummer make the peak of isoprene production in spring. These results can be clearly understood by distinguishing immediate impacts and the impacts of future expectations.

## Introduction

Trees produce a diversity of volatile organic compounds (abbreviated as BVOCs) to protect their leaves from various damages, including heat stress, herbivory, and pathogens^1^. The amount of BVOC production is very large^2^, and the BVOC may modify the local and global climates^3,4^.

BVOCs have protective functions^1^. Isoprene enhanced the heat tolerance of leaves by facilitating recovery after heat stress of leaves through the rapid suppression of reactive oxygen species and stabilizing cell membrane^5–13^. Monoterpenes and sesquiterpenes functions as repellents of herbivorous insects. In addition, trees attacked by herbivorous insects emit BVOCs which attract predatory insects that remove the herbivores^14–18^. In this context, BVOCs functions as infochemicals, calling for predators to guard the trees.

Thanks to the recent rapid advancement of technique in handling genomics and genome-wide gene expression data (e.g., for genes controlling flowering timing of plants^19,20^), information about the genes involved in the production of BVOCs has become increasingly available. For example, both *Quercus glauca* and *Lithocarpus* *edulis* are trees of the *Fagaceae* family living in warm temperate regions of the Japanese islands. In both species, the genes related to isoprene production show high expression in the middle of the summer^21,22^. The gene expression does not necessarily imply the production of respective chemicals, as it is often subject to additional regulatory processes. One possible interpretation is that the tree prepares for the arrival of the season with a high risk of heat stress by activating biochemical pathways needed for producing isoprene, becoming ready to produce the chemical upon the sudden arrival of detrimental heat stress.

In this study, we examine a mathematical model to determine the optimal seasonal pattern of isoprene production for a tree. Isoprene is highly volatile and is not able to stay on leaves, and is able to enhance leaves’ resistance to heat stress. We approach this as an optimal control problem, seeking for the profitable seasonal schedule of isoprene production. The tree is considered as a player aiming to maximize its total net photosynthesis, defined as the total carbon produced by the photosynthesis of a cohort of leaves until their shedding, minus the cost of isoprene production. We assume that the risk of heat stress, photosynthetic rate, and the effectiveness of BVOCs may vary seasonally.

The amount of leaf area changes over time $t$ following a differential equation. If the tree produces more BVOC on day $t$, the leaves are better protected from damages, resulting in improved photosynthesis from the surviving leaves. Whether this benefit exceeds the cost of producing the BVOC depends on various processes. Especially it depends on the day $t$. If $t$ is close to the end of the growing season, the leaves do not have a high expectation of photosynthesis. and hence the value would be small. In contrast, in the early portion of the growing season, the same amount of leaf area would have a greater future prospect simply because the length of time from day $t$ to the end of the season is longer. Hence, the isoprene production would be more profitable in the early portion of a season.

There are numerous processes influencing the benefit of isoprene production. To integrating them systematically, we employ a mathematical method developed in control theory, called Pontryagin’s maximum principle^23,24^. This method has been adopted to elucidate the adaptive responses of organisms to environmental changes, explaining phenomena such as the timing of flowering within a year^25–30^, the balance of multiple organs of individual plants, such as shoot/root balance^31^, as well as in animal life history^32–34^. Additionally, it has been applied to growth of the dwarf males in barnacles^35^, production of shells for protecting mollusks from predation^36^, and the age-dependent concentration of defense chemicals, such as alkaloid, in biennial plants based on leaf age^37^.

## Model

We consider a deciduous tree which produces a cohort of leaves synchronously on date $t=0$ (leaf flushing). The leaves are gradually lost due to physical and biological damages. All remaining leaves are discarded at the end of the growing season ($t=T$). Let $L\left(t\right)$ be the leaf area on day $t$. The dynamics of the leaf area are given as follows:

1 $$\frac{dL}{{dt}} = - \left( {u + \frac{h\left( t \right)}{{1 + b\left( t \right)s\left( t \right)}}} \right)L\left( t \right),$$

for $0<t<T$. The initial value is $L\left(0\right)={L}_{0}$. The leaf area decreases due to random removal at rate $u$ and the risk factor given by heat stress or herbivory $h\left(t\right)$. The intensity of the latter may vary over the season. The plant can reduce the risk by investing in BVOCs. We here consider a highly volatile compound, such as isoprene. Isoprene enhances the speed of removing harmful reactive oxygen species formed in leaves under the heat stress and helps the heat resistance of the leaves^5–10^. We consider the case in which the leaves do not maintain a large amount of isoprene for a prolonged period, assuming that all the isoprene produced on a day would be released within the same day and would not be carried over to the subsequent days, unlike terpenoids in *Lamiacea* or *Pinacea* species^38^. In Eq. (1), $s\left(t\right)$ represents the amount of isoprene per leaf area produced on day $t$. In Eq. (1), the risk of leaf loss is reduced by factor $1/\left(1+b\left(t\right)s\left(t\right)\right)$, and the daily exponential rate of leaf area loss is $u+h\left(t\right)/\left(1+b\left(t\right)s\left(t\right)\right)$.

The objective function is to maximize the net photosynthesis of a cohort of leaves over the whole season, given as follows:

2 $$\phi = \mathop \smallint \limits_{0}^{T} \left( {p\left( t \right) - s\left( t \right)} \right)L\left( t \right)dt,$$

Here *p(t)* represents the photosynthetic rate per unit leaf area, measured in terms of carbon gain. Equation (2) is the total amount of photosynthesis minus the investment in isoprene production. The amount of isoprene produced *s(t)* is measured in terms of the associated cost: the reduction of net CO_2_ assimilation known to be caused by the higher allocation of leaf internal C sources (xylem sugars, starch, cytosolic sources) for isoprene biosynthesis^11^.

This is a typical optimal control problem. The objective function is $\phi$ given in Eq. (2) and the control variable is $s\left(t\right)$ for $0<t<T$, subject to the constraint $0\le s\left(t\right)\le {s}_{max}$, where ${s}_{max}$ is the maximum rate of isoprene production per leaf area per day. Differential equation given by Eq. (1) is another constraint. We search for the optimal schedule of the isoprene production that maximizes $\phi$.

### Pontryagin’s maximum principle

To apply Pontryagin’s maximum principle to this optimal control problem, we define the Hamiltonian as follows:

3 $$H = \left( {p\left( t \right) - s\left( t \right)} \right)L\left( t \right) + \lambda \left( t \right)\left( { - 1} \right)\left( {u + \frac{h\left( t \right)}{{1 + b\left( t \right)s\left( t \right)}}} \right)L\left( t \right),$$

Here, $\lambda \left(t\right)$ is a costate variable corresponding to the leaf area $L\left(t\right)$. Intuitively speaking, $\lambda \left(t\right)$ implies the impact of a unit leaf area on day $t$ in enhancing the net photosynthesis $\phi$, as defined by Eq. (2)^24,31,39^. The costate variable follows the differential equation given by,

4 $$\frac{d\lambda }{{dt}} = - \frac{\partial H}{{\partial L}} = - \left( {p\left( t \right) - s\left( t \right)} \right) + \lambda \left( t \right)\left( {u + \frac{h\left( t \right)}{{1 + b\left( t \right)s\left( t \right)}}} \right)$$

We solve this equation in a backward manner, starting from the terminal condition specified as follows: $\lambda \left(T\right)=0$.

The maximum principle dictates that the Hamiltonian must be maximized at each time $t$ by selecting the control variable $s\left(t\right)$. Hamiltonian given in Eq. (3) is a function of $s\left(t\right)$ with a negative second derivative. According to the calculation in section A.1 of S1 Appendix A, we can derive that the optimal value maximizing the Hamiltonian given in Eq. (3) is equal to $s\left(t\right)=\widehat{s}\left[\lambda \left(t\right),t\right]$, where $\widehat{s}\left[\lambda \left(t\right),t\right]$ is the quantity given as follows:

5a $$\hat{s}\left[ {\lambda \left( t \right),t} \right] = 0,\quad{\text{if }}\lambda \left( t \right) \le \frac{1}{b\left( t \right)h\left( t \right)}$$

5b $$\hat{s}\left[ {\lambda \left( t \right),t} \right] = \frac{1}{b\left( t \right)}\left\{ {\sqrt {\lambda \left( t \right)b\left( t \right)h\left( t \right)} - 1} \right\}, \quad{\text{if }}\frac{1}{b\left( t \right)h\left( t \right)} < \lambda \left( t \right) < \frac{{1 + b\left( t \right)s_{max} }}{b\left( t \right)h\left( t \right)}$$

5c $$\hat{s}\left[ {\lambda \left( t \right),t} \right] = s_{max} ,\quad{\text{if }}\lambda \left( t \right) \ge \frac{{1 + b\left( t \right)s_{max} }}{b\left( t \right)h\left( t \right)}$$

Equation (5) presents the optimal isoprene production rate, $s\left(t\right)$, as a function of $\lambda \left(t\right)$.

To numerically obtain $\lambda \left(t\right)$, we can solve the differential equation given by Eq. (4) by integrating it from the end of the season, with the terminal condition $\lambda \left(T\right)=0$. At each time point $t$, we utilize $s\left(t\right)=\widehat{s}\left[\lambda \left(t\right),t\right]$, as specified in Eq. (5). Refer to section A.2 of S1 Appendix A for a detailed derivation of the solution $\lambda \left(t\right)$ including numerical procedure.

**Figure 1:**
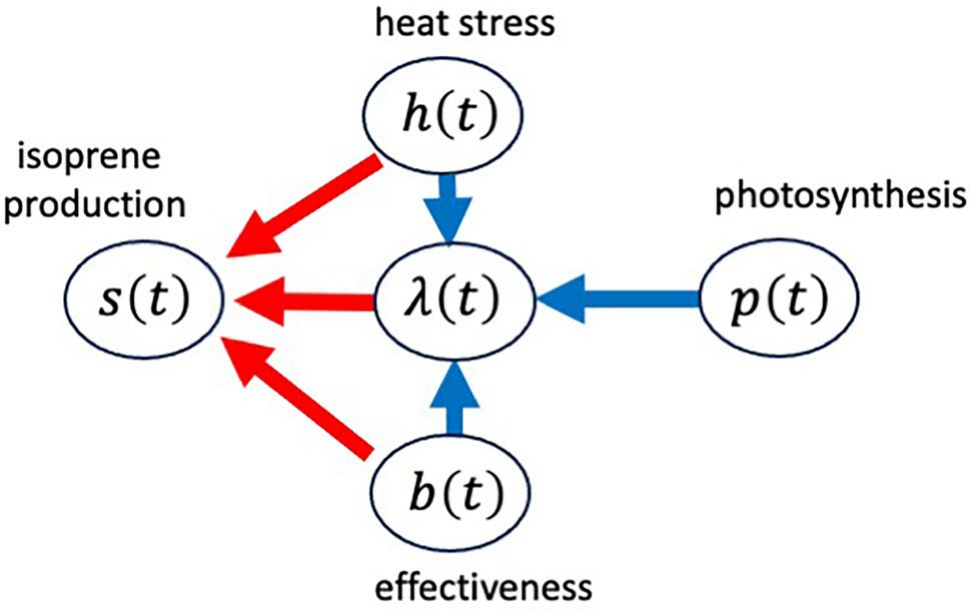
Scheme of how the optimal isoprene production is affected by time-dependent rates. The optimal isoprene production rate $s\left(t\right)$ is determined by heat stress $h\left(t\right)$, the effectiveness of BVOC $b\left(t\right)$, and the marginal value of leaf area $\lambda \left(t\right)$ on the same day. These immediate impacts are indicated by red arrows. Additionally, $s\left(t\right)$ is influenced by future values of $p\left(t\right), h\left(t\right)$, and $b\left(t\right)$, which modify $\lambda \left(t\right)$ on day $t$. These impacts of future expectations are indicated by blue arrows. In the text, we refer to these influences as direct and indirect effects, respectively.

In the subsequent sections, we discuss how the optimal schedule of isoprene production depends on different parameters and rate functions included in the model. Understanding the results involves noting the dependence of isoprene production rate $s\left(t\right)$ in the optimal schedule on various rate functions, as summarized in Fig. 1.

First, Eq. (5) indicates that the isoprene production rate $s\left(t\right)$ is determined by strength of heat stress $h\left(t\right)$, the effectiveness of isoprene in suppressing stress $b\left(t\right)$, and the marginal value of leaf area $\lambda \left(t\right)$ on the same day $t$. This implies that the changes in these quantities affect the isoprene production rate immediately, which may be called “immediate impacts.” In Fig. 1, this is indicated by three red arrows from $h\left(t\right)$, $b\left(t\right)$, and $\lambda \left(t\right)$ to $s\left(t\right)$, respectively.

Second, $\lambda \left(t\right)$ is obtained from integrating differential equation given by Eq. (4) from $t$ to the end of the season $T$. Hence, $\lambda \left(t\right)$ depends on the photosynthetic rate, heat stress, effectiveness of BOVC after $t$ (i.e., $p\left(t{\prime}\right)$, $h\left(t{\prime}\right)$ and $b\left(t{\prime}\right)$ for $t<t{\prime}<T$). For example, an enhancement of photosynthestic rate $p\left(t{\prime}\right)$ on day $t{\prime}$ affects the value of leaf area $\lambda \left(t\right)$ on an earlier date $t$ (i.e.,$t<t{\prime}$). This indicates that the value of leaf area $\lambda \left(t\right)$ depends on the expected value of photosynthetic rate in the future. We may call this as “the impact of future expectations.” In Fig. 1, this is indicated by a blue arrow from $p\left(t\right)$ to $\lambda \left(t\right)$.

Photosynthetic rate $p\left(t\right)$ does not affect $s\left(t\right)$ directly, but it affects $s\left(t\right)$ indirectly via modifying marginal value $\lambda \left(t\right)$. In Fig. 1, this is shown by a blue arrow from $p\left(t\right)$ to $\lambda \left(t\right)$ together with a red arrow from $\lambda \left(t\right)$ to $s\left(t\right)$. Both $h\left(t\right)$ and $b\left(t\right)$ exert direct and indirect effects (i.e., immediate impacts and the impacts of future expectations) on the optimal isoprene production $s\left(t\right)$.

In section A.3 of S1 Appendix A we derive the differential equation Eq. (4) only from the definition of $\lambda \left(t\right)$ as the value of unit leaf area on day $t$. We also justify the argument that the optimal value of isoprene production rate is given by Eq. (5) without resorting the official mathematical formalism of the Hamiltonian.

## Optimal seasonal schedule

Based on the method explained above, we generated the optimal seasonal schedule.

### Final phase without investing isoprene production

As a season progresses, the marginal value of leaf area $\lambda \left(t\right)$ decreases following Eq. (5) and reaches zero at $t=T$. Toward the end of the growing season, there is a period during which no isoprene is produced. This is because protecting leaves from damage is not profitable when the number of remaining days is small. Let ${t}_{s}$ be the date at which investment to isoprene production ceases. We integrate Eq. (4) with $s\left(t\right)$ replaced by $\widehat{s}\left[\lambda \left(t\right),t\right]$ in a backward manner from the final date $T$ with $\lambda \left(T\right)=0$. From $\lambda \left({t}_{s}\right)b\left({t}_{s}\right)h\left({t}_{s}\right)=1$, we have the following condition for ${t}_{s}$.

6 $$\frac{1}{{b(t_{s} )h(t_{s} )}} = \int\limits_{{t_{s} }}^{T} {p(t^{\prime } )} \exp \left[ { - \int\limits_{{t_{s} }}^{{t^{\prime } }} {(u + h(t^{\prime \prime } ))dt^{\prime \prime } } } \right]dt^{\prime }$$

When photosynthesis rate is slow, the isoprene is not effective in suppressing stress, or heat stress is small, the isoprene production is not profitable to the tree. Equation (6) is not satisfied, because the left-hand side of Eq. (6) is larger than the right-hand side for all ${t}_{s}$.

When all the rates are independent of time, Eq. (6) specifies the onset of the final phase of no isoprene production as follows:

7 $$t_{s} = T - \frac{1}{u + h}ln\left( {\frac{1}{{1 - \frac{u + h}{{pbh}}}}} \right)$$

The derivation is explained in S1 Appendix A. Equation (7) indicates that the isoprene production lasts longer if the photosynthesis is fast (large $p$), heat stress is strong (large $h$), and isoprene is effective (large $b$).

### When all the rates are independent of time t

**Figure 2:**
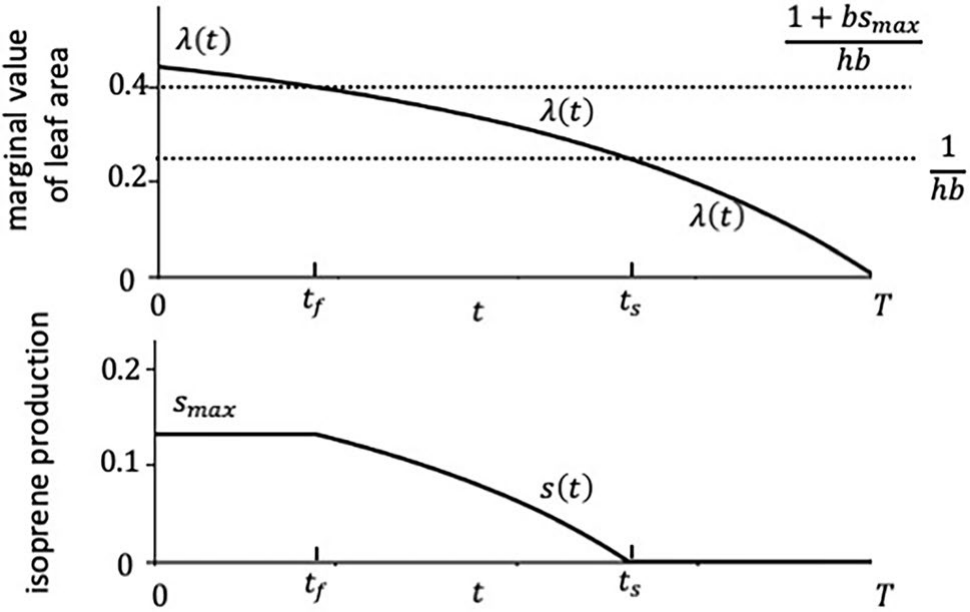
Optimal schedule when all parameters are constant. The BVOC is produced at the maximum rate $s\left(t\right)={s}_{max}$ in the first period, an intermediate rate $0<s\left(t\right)<{s}_{max}$ is the second period, and no BVOC is produced in the final period $s\left(t\right)=0$. Parameters are: $p=1$, $h=2$, $b=2$, $u=0.0001$, $T=1$, ${s}_{max}=0.132$.

First, we consider simple situations in which all rates (photosynthesis $p\left(t\right)$, harm $h\left(t\right)$, and effectiveness of isoprene $b\left(t\right)$) are independent of time, $t$. Figure 2 illustrates a typical case in which there are three phases. In the first phase, just after leaf flush, isoprene production occurs at the fastest rate. This period is followed by the second phase, during which isoprene is produced at an intermediate rate (between 0 and ${s}_{max}$). After ${t}_{s}$, there is a final period in which no isoprene is produced. The switching time ${t}_{s}$ is given by Eq. (7).

The level of isoprene production is also influenced by parameters within the model. For example, while the seasonal pattern of the isoprene production $s\left(t\right)$ remains consistent, an increase in the photosynthetic rate results in a higher $s\left(t\right)$ value and maintains positivity for an extended duration within the season (refer to Fig. S1 in SI Appendix B for detail). In SI Appendix B, we explain the results of parameter dependence in detail. When ${s}_{max}$ is sufficiently large, the optimal schedule does not include Phase I, indicating that isoprene production is not constrained by the maximum daily production rate. Then, the maximum rate of isoprene production is $s\left(0\right)$. The elasticities of $s\left(0\right)$ with respect to $p$, $h$, $b$, and $T$ were positive and of a similar order of magnitude, although their precise values vary depending on the choice of the standard parameter sets. In contrast, the elasticity with respect to $u$ was negative and significantly smaller in magnitude than the others. From these results, we concluded that the optimal isoprene production tends to increase with a higher photosynthesis rate, increased heat stress, improved efficiency of the chemical, and a longer growing season.

### When harm h(t) peaks in the summer

**Figure 3:**
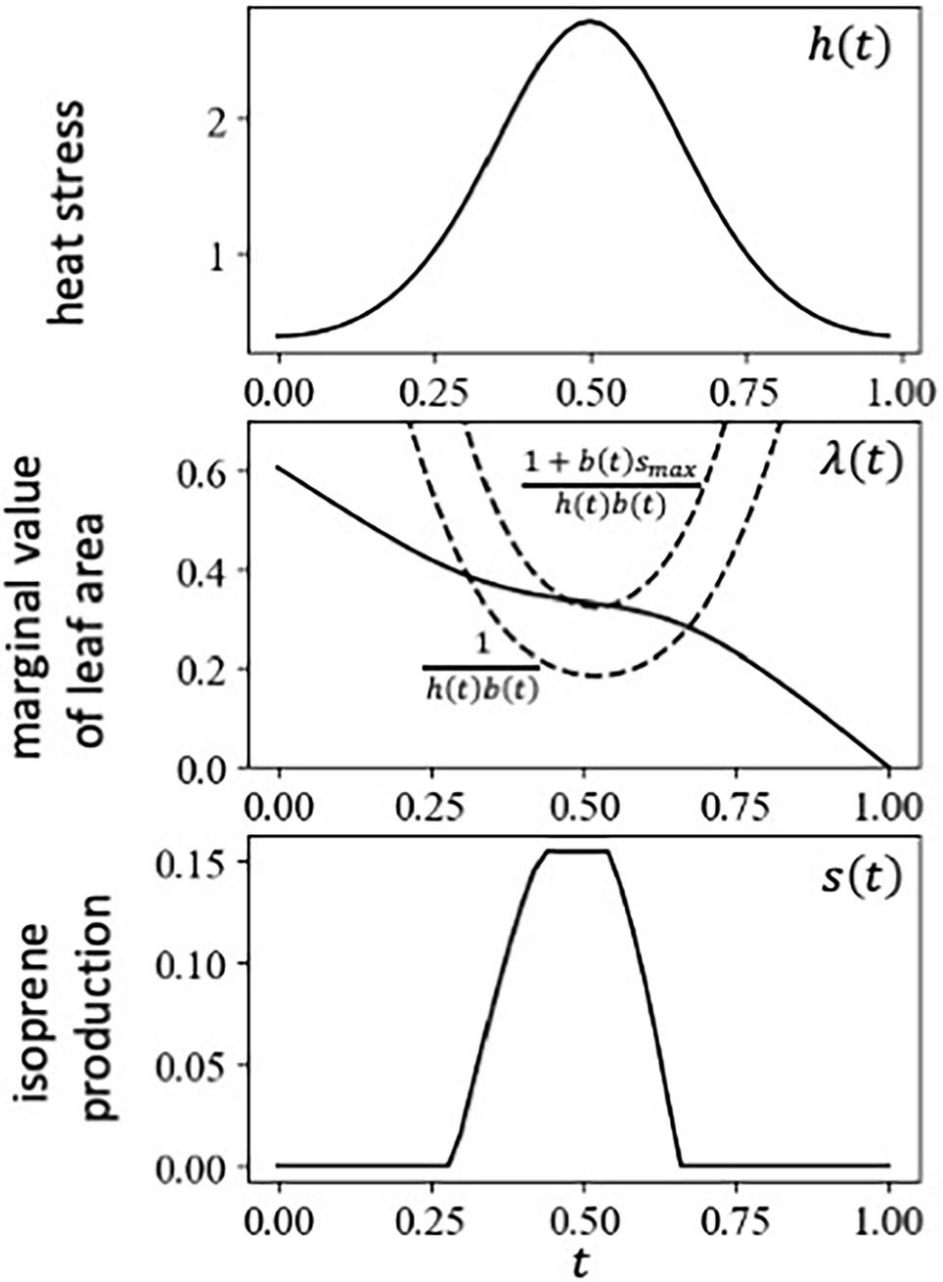
Optimal schedule when heat stress $h\left(t\right)$ has a peak in midsummer. $h\left(t\right)=exp\left[{a}_{1}-{b}_{1}cos\left(\frac{2\pi t}{T}\right)\right]$, where ${a}_{1}=0$, ${b}_{1}=1$, and $T=1$. Other rates are constant: $p=1$, $b=2$, $u=0.0001$, ${s}_{max}=0.155$. Isoprene production $s\left(t\right)$ is high in midsummer when heat stress is strong.

Heat stress is expected to be more pronounced in the summer than in spring or fall. Figure 3 illustrates the cases where $h\left(t\right)$ reaches its peak in the middle of the growing season. Isoprene production is low or small in the spring or early summer, increases to a peak level in the mid-summer, and decreases in the late summer. The heat stress is an exponentially modulated cosine function, $h\left(t\right)=exp\left[a-b cos\left\{2\pi t/T\right\}\right]$, which attains the maximum $exp\left[a+b\right]$ at $t=T/2$ and the minimum $exp\left[a-b\right]$ at $t=0$ and $t=T$. A larger $b$ indicates a sharper peak of heat stress. We may call $b$ as “shape factor.”

**Figure 4:**
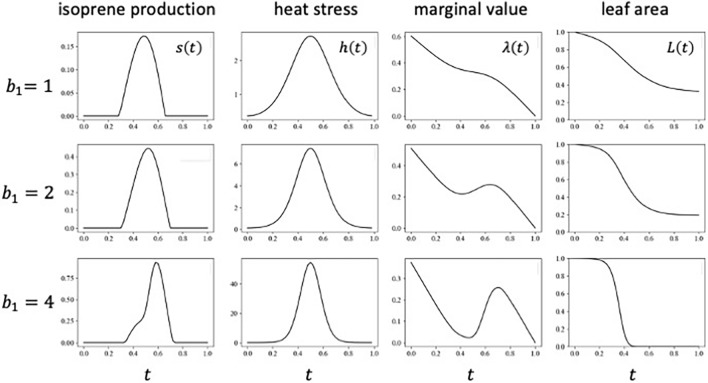
Optimal schedule of isoprene production when heat stress has a peak in the middle of the season (i.e. summer). We adopted an exponentially modulated cosine function, $h\left(t\right)=exp\left[{a}_{1}-{b}_{1}cos\left(\frac{2\pi t}{T}\right)\right]$. All other parameters are constant. We fixed ${a}_{1}=0$. Three cases differ in ${b}_{1}$, which changes ${H}_{total}$ (${b}_{1}=1$, $2$, and $4$, respectively in top, middle, and bottom parts). As $a$ increases, the total stress increases (${H}_{total}=1.27$, $2.28$, and $11.3$, respectively). Isoprene production peaks slightly later than the peak heat stress, with the magnitude of their difference increasing with the total stress. Other parameters are: $p=1$, $b=2$, $u=0.0001$, $T=1$.

- (i) High isoprene production during the period of high heat stress
  Figure 4 shows a numerical example of the optimal schedule of isoprene production, exhibiting three different values of strength of heat stress. The optimal isoprene production had a peak in the mid-summer. This is plausible because isoprene functions to cope with heat stress in the mid-summer. The three parts are for cases with the same shape constant $b$ but different $a$. As $a$ increases, the total heat stress t ${H}_{total}= {\int }_{0}^{T}h\left(t\right)dt$ increases, and the peak date of isoprene production remained near the peak date of heat stress (midsummer).
- (ii) Post-risk enhancement
  However, if $s\left(t\right)$ curve is closely examined, the date of the highest rate of isoprene production occurred a little later than the peak of the heat stress. The difference between the two peaks became more pronounced for a larger total heat stress (Fig. 4). This suggests that the isoprene is produced at the maximum rate in late summer or even in early autumn. The reason is explained by the non-monotonic shape of the marginal value of leaf area $\lambda \left(t\right)$. When the heat stress was very strong, $\lambda \left(t\right)$ became the lowest before the mid-summer of heat stress and increased rapidly in the high-risk period, resulting in a rather high value of leaf area in the late summer or autumn, as shown in Fig. 4. The marginal value of leaf area exhibited this behavior because leaf area sampled before the high-risk period was more likely to be removed during that time, rendering the leaf area sampled after the risky period more value than that sampled before it. We call this increase of the value as "post-risk enhancement." The magnitude of this effect was related to the amount of leaf areas loss in this peak stress, observed in the decrease in $L\left(t\right)$.
  In SI Appendix C, we explain the results for different constant $b$ for the shape of $h\left(t\right)$, with the total heat risk ${H}_{total}= {\int }_{0}^{T}h\left(t\right)dt$ controlled, which suggest that the magnitude of the peak shift was more strongly controlled by total heat risk ${H}_{total}$ than by shape constant $b$.

### Effects of seasonal changes in photosynthesis and volatility

We also examined the effect of seasonal change in photosynthetic rate of leaves $p\left(t\right)$ and the effectiveness of isoprene in suppressing heat stress $b\left(t\right)$.

**Figure 5:**
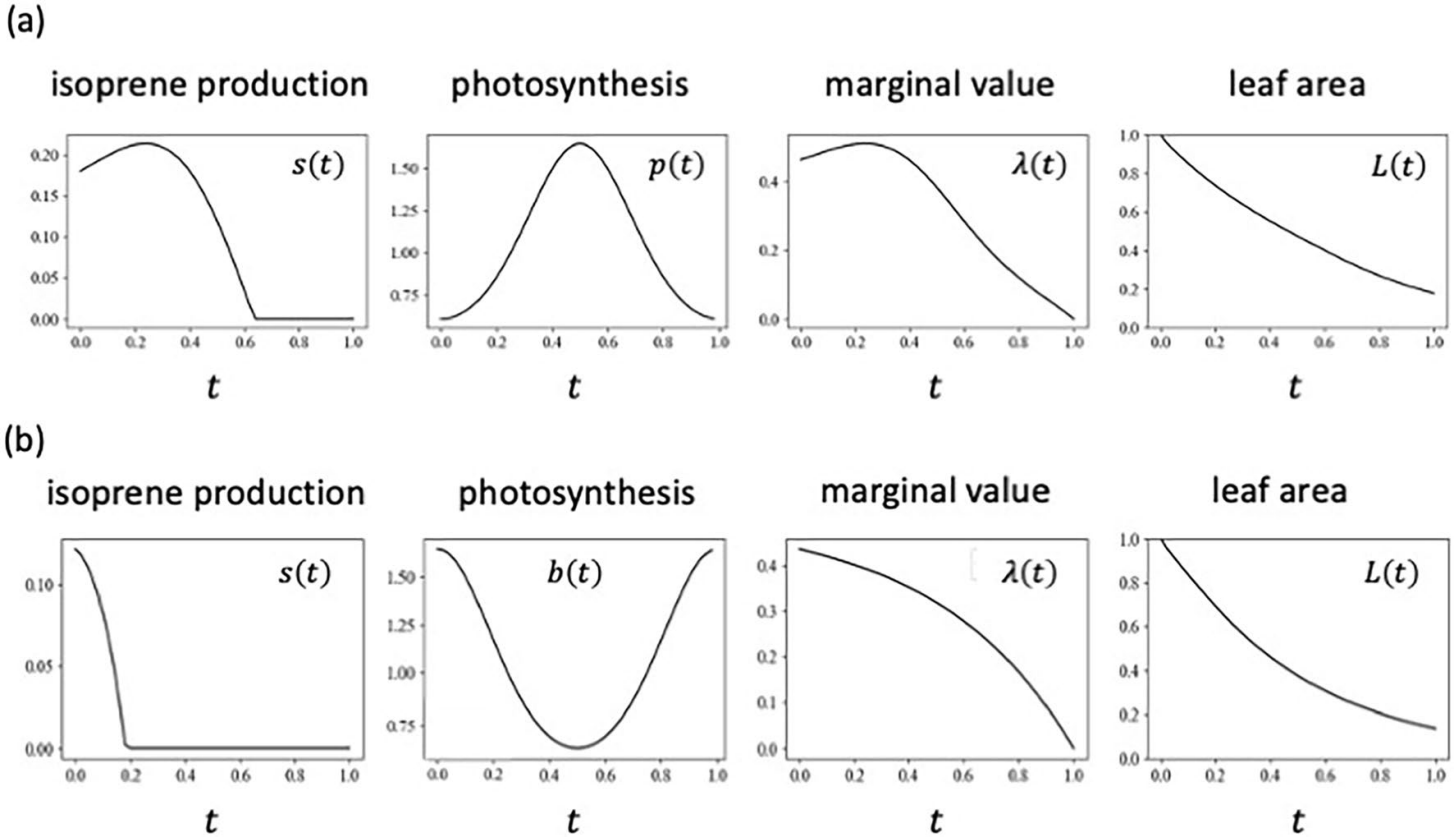
Optimal schedules of isoprene production. (**a**) When photosynthesis rate $p\left(t\right)$ has a peak in midsummer. $p\left(t\right)=exp\left[{a}_{1}-{b}_{1}cos\left(\frac{2\pi t}{T}\right)\right]$ with ${a}_{1}=0$, ${b}_{1}=0.5$. The isoprene production has a peak in the spring. (**b**) When effectiveness of BVOC $b\left(t\right)$ drops in midsummer. $b\left(t\right)=0.5exp\left[{a}_{1}+{b}_{1}cos\left(\frac{2\pi t}{T}\right)\right]$, with ${a}_{1}=0$, ${b}_{1}=0.5$. $s\left(t\right)$ has a peak in the spring, and no BVOC is produced in the summer or autumn. Other parameters are: $h=2$, $b=2$, $u=0.0001$, $T=1$.

Figure 5(a) illustrates the results when $p\left(t\right)$ has a peak in midsummer, with other rates constant. The optimal isoprene production reaches its peak in spring or in early summer and decreases sharply in midsummer and autumn. This result can be understood from the pattern of marginal value of leaf area $\lambda \left(t\right)$, which declines rapidly in summer. As shown in Fig. 1, photosynthetic rate $p\left(t\right)$ affects the isoprene production $s\left(t\right)$ indirectly via modifying the marginal value $\lambda \left(t\right)$. A high peak $p\left(t\right)$ in midsummer leads to a high peak of $\lambda \left(t\right)$ in spring and the decrease of $\lambda \left(t\right)$ and $s\left(t\right)$ in summer, because $p\left(t\right)$ has only the impact of future expectations on $s\left(t\right)$ (see Fig. 1).

In midsummer, the volatility of the chemicals may increase due to high temperatures, leading to a drop of $b\left(t\right)$ in midsummer. Figure 5(b) illustrates the effect of this seasonality. The optimal isoprene production is high in spring or in early summer, but it becomes zero in midsummer and in autumn. $b\left(t\right)$ has both direct and indirect effects on $s\left(t\right)$ (see Fig. 1). The drop of $b\left(t\right)$ in midsummer would directly reduce $s\left(t\right)$ in summer (immediate impacts). Furthermore, the indirect effect of $b\left(t\right)$ drop causes a rapid decline in $\lambda \left(t\right)$ (impacts of future expectations). The combination of these effects may stop isoprene production in midsummer and autumn, leading to the peak of $s\left(t\right)$ in spring.

## Discussion

Many trees produce and emit biogenic volatile organic compounds (BVOCs), which protect their leaves from various damages, including herbivory, pathogens, and heat stress^1^. The amount of isoprene production by trees is very large^2^, and BVOCs may modify the local and global climates^3,4,40^. Studies of the molecular and physiological mechanisms have also been advanced^21,41–46^. Understanding the seasonal pattern of gene expression would be helpful in revealing the mechanism controlling genes, considering the respective roles of their products.

In this study, to understand the adaptation of different seasonal patterns of isoprene production, we analyzed the optimal seasonal schedule for producing highly volatile BVOCs, such as isoprene, in leaves that mitigate damage caused by heat stress^5–9,13^. We formalized a dynamic optimization problem for the seasonal schedule of Isoprene production. Using Pontryagin's maximum principle, we searched for the optimal seasonal schedule that maximizes the total net photosynthesis and examined how it varies with seasonal patterns of photosynthetic rate, heat stress, and the stress-suppressing effect of isoprene.

### Isoprene production level depends on future expectations

Producing isoprene provides benefits to the tree through increased resistance to heat stress and elevated defense levels against herbivores/pathogens. Since the benefit arises from the preservation of leaves, it depends on the length of the period for them to perform photosynthesis. Toward the end of the growing season, the tree makes no BVOC because the benefit is smaller than the cost. If the time until the end of the season is sufficiently long, producing some isoprene becomes beneficial. To represent how the value of preservation of leaves changes with time in the growing season, marginal value of leaf area $\lambda \left(t\right)$ is defined. In section A.4 of SI Appendix A, we derive that $\lambda \left(t\right)$ is equal to the total net production to be made until the end of the season per leaf area.

The second aspect important in evaluating the benefit of additional isoprene production is that the effect of stress suppression by additional amount diminishes as stress is reduced by the chemical already produced. The benefit of isoprene production of an additional unit amount is the effect of suppressing leaf area loss multiplied by the marginal value of leaf area. Maintaining a production rate that strikes the balance between the benefits and costs of isoprene production provides the optimal solution.

We may ask the amount and seasonal pattern of isoprene production that is the most profitable to the tree. In simple cases where the photosynthetic rate, stress intensity, and the stress-mitigating effect of isoprene are all constant, isoprene production should be high in the early growing season, decreasing gradually and reaching zero toward the end of the season, as shown in Fig. 3. According to elasticity analysis, trees tend to produce more isoprene in highly productive environments, under increased heat stress, with more effective inhibition by isoprene, during a longer growing season, and with a smaller leaf loss rate.

### Seasonal changes of different processes shape the isoprene seasonal pattern

Next, we investigated the optimal isoprene production schedule in under seasonal changes. When heat stress $h\left(t\right)$ peaks in the mid-summer with others constant, optimal isoprene production has a peak in midsummer, as shown in Figs. 4, S1. This result is plausible.

The bottom parts of Fig. 5 illustrated the results when the total amount of stress ${\int }_{0}^{T}h\left(t\right)dt$ was large. The maximum rate of isoprene production per unit leaf area occurs later than the peak of heat stress $h\left(t\right)$, namely, late summer or autumn, rather than midsummer. This counter-intuitive result is explained by the nonmonotonic seasonal change in the marginal value of leaf area: $\lambda \left(t\right)$ is small just before the peak heat stress. It rises sharply during the mid-summer and has the maximum after the peak of $h\left(t\right)$. The direct effect of seasonal change of $h\left(t\right)$ makes $s\left(t\right)$ high in mid-summer, while the indirect effect of $h\left(t\right)$ causes the delay in the peak $s\left(t\right)$. The seasonal pattern of $s\left(t\right)$ is the combined result of these two effects (see Fig. 1). We called the increase in $\lambda \left(t\right)$ during the time of a strong heat stress “post-risk enhancement” of the marginal value of leaf area. This effect is pronounced only when a substantial fraction of leaves is lost during the period. While having peak activity in late summer seems counter intuitive, but it is interesting to note that Mayrhofer et al.^41^ reported in grey popular leaves that the protein concentration and enzymatic activity of Isoprene synthase (PcISPS) peaked in late summer, while transcriptional levels of *PcISPS* gene were the highest in early summer.

When photosynthetic rate $p\left(t\right)$ peaks in midsummer with other rates constant, the optimal isoprene production has a peak in spring. The summer peak of $p\left(t\right)$ makes the marginal value of leaf area $\lambda \left(t\right)$ decline quickly in midsummer and becomes small in autumn. Since $p\left(t\right)$ has no immediate impacts on $s\left(t\right)$ (see Fig. 1), $s\left(t\right)$ has a peak in spring. In a similar manner, when the effectiveness of the isoprene in mitigating heat stress $b\left(t\right)$ drops in midsummer due to the high volatility, the optimal isoprene production has a peak in spring. These are not the pattern observed for genes involved in the pathway for production of isoprene^21^. Hence, we may conclude that the simplest way to explain the peak isoprene production in midsummer is the high heat stress. The seasonal changes in photosynthetic rate or volatility play a less significant role.

To comprehend how the seasonality of different processes shapes the optimal isoprene production pattern, we need to distinguish between the immediate impacts and those stemming from future expectations (refer to Fig. 1).

### Future problems

There are several different ways of future theoretical studies extending the model examined in this paper.

- (i) mildly volatile BVOC
  In a study of genome-wide gene expression data, focusing on *Quercus glauca* and *Lithocarpus* *edulis* in warm temperate regions of Japanese islands, genes related to sesquiterpene production were highly expressed in spring, while genes related to isoprene production exhibited high expression in the middle of the summer^21,22^. Sesquiterpene is much less volatile than isoprene. The observed difference in the seasonal pattern of gene expression could be attributed to the difference in the turnover rate of the respective chemicals. To address this hypothesis, we may consider the optimal seasonal schedule of producing BVOC with mild (or low) volatility. We conjecture that the most efficient seasonal schedule could involve concentrating the production of most chemicals at the beginning of the growing season, without additional production in the rest of the year.
  To be specific, we may assume that the risk is reduced by the presence of BVOC per leaf area $V\left(t\right)$, as $h\left(t\right)/\left(1+aV\left(t\right)\right)$. The amount of BVOC on day $t$ is not proportional to the production on the same day but it is determined by the amount produced on or before the day. For example, $V\left(t\right)$ might follow a differential equation, $dV/dt=s\left(t\right)-k\left(t\right)V\left(t\right)$, where $s\left(t\right)$ is the production rate of BVOC. The model is more challenging to analyze than the one discussed in the present study due to the presence of two differential equations in the system. It is a problem worthy of being addressed in the future studies.
- (ii) BVOC emission as communication between and within individuals
  Plants under stress might benefit from signaling to other individuals. For instance, when one tree is attacked by herbivores, it may release monoterpenes and sesquiterpenes and attract predatory insects, which help in eliminating the herbivorous insects^14–18^. Additionally, when a tree is under attack, it may emit defence-related BVOCs, makes the recipient trees start producing the BVOCs. This could induce a form of defense in nearby trees, known as “induced systemic resistance”^47^. Furthermore, when a branch is attacked by herbivores and emits BVOCs, other branches of the same individual initiate the production of the BVOCs, thereby enhancing the overall the defense level. The diverse aspects of communication, among different species, among individuals of the same species, and within a single individual, will be an important subject of study in theoretical biology.
- (iii) Alternative method of calculating optimal control
  In the present study, Pontryagin's maximum principle was adopted for analysis. Dynamic programming is an alternative method of solving the dynamic optimization problem^48,49^, which has been used in the evolutionary ecology to analyze the optimal life history schedule of plants and animals^39,50–52^. Since dynamic programming easily extends to situations including discrete-time dynamics and stochastic environments, developing corresponding analyses based on the dynamic programming method may provide us with a different perspective on the problem.
- (iv) Mild heat stress reducing the photosynthetic rate in a reversible manner
  A severe heat stress is known to damage the photosynthetic machinery in an irreversible manner^12^, which can be effectively modeled as the loss of leaf area as in this paper. However, mild heat stress is known to reduce the photosynthetic rate in a reversible manner^12^. To model such situations, we need to consider the possibility that the photosynthetic rate can be reduced by the heat, which may also be mitigated by isoprene production. For example, $p\left(t\right)$ in Eq. (2) is replaced by $p\left(t\right)exp\left[-\gamma h\left(t\right)/\left(1+b\left(t\right)s\left(t\right)\right)\right]$. In this altered situation, the optimal isoprene production $s\left(t\right)$ may depend on $p\left(t\right)$. This is an important theme for future studies.
  The model studied in this paper was chosen to be the simplest. We may extend it in several different directions, incorporating various complexities, such as unpredictable environmental fluctuations like irregular heatwaves, pest outbreaks, or weather patterns, and interactions with a broader ecosystem. Additionally, we will consider the resilience and adaptability of the BVOC production strategy and the potential feedback loops between BVOC emissions and climate change. This includes examining both the local cooling effects (due to aerosol formation) and the global warming potential (due to methane and ozone formation).

### Supplementary Information

Supplementary Information.

## Data Availability

All data generated or analysed during this study are included in this published article and its supplementary information files.
