# Supplementary material for: Optimal seasonal schedule for the production of isoprene, a highly volatile biogenic VOC
Source: Sci Rep. 2024 May 29;14:12311. doi: 10.1038/s41598-024-62975-3 (PMC11137007; doi:10.1038/s41598-024-62975-3)
Supplement: Supplementary file 1 — Supplementary Information. [file 41598_2024_62975_MOESM1_ESM.docx]

**SUPPLEMENTARY INFORMATION**

**(Appendices A-C)**

**Optimal seasonal schedule for the production of isoprene,**

**a highly volatile biogenic VOC**

**Yoh Iwasa, Rena Hayashi, and Akiko Satake**

**Appendix A**

**Derivation of the optimal solution in Pontryagin's maximum principle**

Here, we derive the optimal schedule based on Pontryagin's maximum principle.

**A.1 *Intuitive derivation of differential equation for*** $\boldsymbol{\lambda}\left( \boldsymbol{t} \right)$

According to Pontryagin's maximum principle, we can derive the differential equation of $\lambda\left( t \right)$ from the Hamiltonian, as indicated by Eq. (4) in the main text. Here we explain an intuitive derivation of the same differential equation based on the definition of $\lambda\left( t \right)$ as the value of unit leaf area on day $t$, without resorting the formal argument using the Hamiltonian.

$\lambda\left( t \right)$ is defined as the value of leaf are, or the future total net gain to be made from $t$ to $T$ per unit leaf area. Then the total net gain of the whole cohort of leaves is $\lambda\left( t \right)L\left( t \right)$. We separate the carbon gain made within a short time interval of length $\Delta t$ and the net gain to be made after the interval, namely from $t+\Delta t$ to $T$. We have following equation:

$\lambda\left( t \right)L\left( t \right)=\left( p\left( t \right)-s\left( t \right) \right)L\left( t \right)\Delta t+\lambda\left( t+\Delta t \right)L\left( t+\Delta t \right)$ (A.1)

where we neglect small terms of order of $o\left( \Delta t \right)$. Using the differential equation for ${dL}/{dt}$ as Eq. (1), the second term in the right-hand side is rewritten as follows:

$$\lambda\left( t \right)L\left( t \right)=\left( p\left( t \right)-s\left( t \right) \right)L\left( t \right)\Delta t+\lambda\left( t+\Delta t \right)\left[ L\left( t \right)-\left( u+\frac{h\left( t \right)}{1+b\left( t \right)s\left( t \right)} \right)L\left( t \right)\Delta t \right]$$

(A.2)

We rearrange terms and obtain the following:

$$\left( -1 \right)\frac{\lambda\left( t+\Delta t \right)-\lambda\left( t \right)}{\Delta t}L\left( t \right)=\left( p\left( t \right)-s\left( t \right) \right)L\left( t \right)-\lambda\left( t \right)\left( u+\frac{h\left( t \right)}{1+b\left( t \right)s\left( t \right)} \right)L\left( t \right)$$

In the limit of $\Delta t\to0$, we have

$\frac{d\lambda}{dt}=-\left( p\left( t \right)-s\left( t \right) \right)+\lambda\left( t \right)\left( u+\frac{h\left( t \right)}{1+b\left( t \right)s\left( t \right)} \right)$ (A.3)

which is the same as Eq. (4) in the text derived from Pontyragin's maximum principle.

In the model studied in this paper, the state variable $L$ follows linear dynamics. When the differential equation for the state variable is nonlinear, we need to consider the effect of a small change in the state variable made at $t$ and trace its effect to the objective function (refer to Iwasa and Roughgarden, 1984). Then, the differential equation for the marginal value of state variable is given by the partial differential equation of the Hamiltonian, as ${d\lambda}/{dt}=-{\partial H}/{\partial L}.$

**A.2 *Optimal level of isoprene production as a function of*** $\boldsymbol{\lambda}\left( \boldsymbol{t} \right)$

We consider the optimal of isoprene production $s\left( t \right)$ as the value which maximizes the contribution to the fitness, as given by Eq. (A.2)

$\left( p\left( t \right)-s\left( t \right) \right)L\left( t \right)\Delta t+\lambda\left( t+\Delta t \right)\left[ L\left( t \right)-\left( u+\frac{h\left( t \right)}{1+b\left( t \right)s\left( t \right)} \right)L\left( t \right)\Delta t \right]$ (A.4)

We choose $s\left( t \right)$ to maximize this quantity. We can see the relationship of this quantity and the Hamiltonian given by Eq. (3) in the text. It depends on $s$ as follows:

$${max}_{0\leq s\leq s_{max}}\left\{ -sL\left( t \right)-\lambda\left( t \right)\frac{h\left( t \right)}{1+b\left( t \right)s}L\left( t \right)+\left[ \begin{aligned} \mathrm{terms} \\ independent of s \end{aligned} \right] \right\}$$

The expression within the curly braces is a function of $s$ with the following first and the second derivatives:

$$\frac{\partial}{\partial s}\left\{ ... \right\}=\left( -1+\lambda\left( t \right)\frac{h\left( t \right)b\left( t \right)}{\left( 1+b\left( t \right)s \right)^{2}} \right)L\left( t \right)$$

$$\frac{\partial^{2}}{\partial s^{2}}\left\{ ... \right\}=-2\frac{\lambda\left( t \right)h\left( t \right){b\left( t \right)}^{2}}{\left( 1+b\left( t \right)s \right)^{3}}<0$$

Let $\hat{s}$ be the value of $s$ achieving the first derivative to be zero:

$s^{*}=\frac{1}{b\left( t \right)}\left( \sqrt{\lambda\left( t \right)h\left( t \right)b\left( t \right)}-1 \right)$ (A.5)

If $s^{*}$ is within the allowed interval $0<s^{*}<s_{max}$, $s^{*}$ is the optimal value of $s$. The optimal value of $s$ is 0 if $\hat{s}\leq0$, and it is $s_{max}$ if $s^{*}\geq s_{max}$. We introduce $\hat{s}\left[ \lambda\left( t \right),t \right]$ as the value of $s$ that maximizes the Hamiltonian, as follows:

$\hat{s}\left[ \lambda\left( t \right),t \right]=0$ , if $\frac{1}{b\left( t \right)}\left( \sqrt{\lambda\left( t \right)h\left( t \right)b\left( t \right)}-1 \right)\leq0$

$\hat{s}\left[ \lambda\left( t \right),t \right]=\frac{1}{b\left( t \right)}\left\{ \sqrt{\lambda\left( t \right)b\left( t \right)h\left( t \right)}-1 \right\}$ , if $0<\frac{1}{b\left( t \right)}\left( \sqrt{\lambda\left( t \right)h\left( t \right)b\left( t \right)}-1 \right)<s_{max}$

$\hat{s}\left[ \lambda\left( t \right),t \right]=s_{max}$ , if $\frac{1}{b\left( t \right)}\left( \sqrt{\lambda\left( t \right)h\left( t \right)b\left( t \right)}-1 \right)\geq s_{max}$

(A.6)

which is rewritten as Eq. (6) in the main text.

**A.3 *Numerical calculation of*** $\boldsymbol{\lambda}\left( \boldsymbol{t} \right)$

The differential equation for costate variable $\lambda\left( t \right)$ is given as Eq. (4). The terminal condition is $\lambda\left( T \right)=0$. We integrate Eq. (4) with respect to time $t$ in a backward manner. The recursive formula for calculating $\lambda\left( t \right)$ for $0<t<T$ is given as follows:

$\lambda\left( t-\Delta t \right)=\lambda\left( t \right)-\Delta t\left\{ -\left( p\left( t \right)-\hat{s}\left[ \lambda\left( t \right),t \right] \right)+ \right.\lambda\left( t \right)\left( u+\frac{h\left( t \right)}{1+b\left( t \right)\hat{s}\left[ \lambda\left( t \right),t \right]} \right)$ (A.7)

where $\hat{s}\left[ \lambda\left( t \right),t \right]$ is given by Eq. (A.5), which is rewritten as Eq. (6) in the text.

**A.4 *Constant environment***

When $p\left( t \right)$, $h\left( t \right)$, and $b\left( t \right)$ are independent of time $t$, Eq. (6) in the text becomes as follows:

$$\frac{1}{bh}=\int_{t_{s}}^{T} p\cdot exp\left[ -\int_{t_{s}}^{t'} \left( u+h \right)dt" \right]dt'$$

The start of the final period without isoprene production is given by the switching date $t_{s}$. The above equation is rewritten as

$$\frac{1}{pbh}=\frac{1}{u+h}\left( 1-e^{-\left( u+h \right)\left( T-t_{s} \right)} \right)$$

which leads to

$T-t_{s}=\frac{-1}{u+h}ln\left( 1-\frac{u+h}{pbh} \right)$ (A.8)

which is rewritten as Eq. (7) in text.

**A.5 Costate variable**

The product of the constant variable $\lambda\left( t \right)$ and the leaf area $L\left( t \right)$. Using Eqs. (1) and (4) in the text, we can derive the following differential equation for their product:

$$\frac{d}{dt}\left( \lambda\left( t \right)L\left( t \right) \right)=\left\{ -\left( p\left( t \right)-s\left( t \right) \right)-\lambda\left( t \right)\left( -1 \right)\left( u+\frac{h\left( t \right)}{1+b\left( t \right)s\left( t \right)} \right) \right\}L\left( t \right)$$

$+\lambda\left( t \right)\left( -1 \right)\left( u+\frac{h\left( t \right)}{1+b\left( t \right)s\left( t \right)} \right)L\left( t \right)$

$=-\left( p\left( t \right)-s\left( t \right) \right)L\left( t \right)$ (A.9)

We note $\lambda\left( T \right)L\left( T \right)=0\cdot L_{o}=0$. By integrating Eq. (A.9)

$\lambda\left( t \right)L\left( t \right)=\int_{t}^{T} \left( p\left( t' \right)-s\left( t' \right) \right)L\left( t' \right)dt'$ (A.10)

which leads to the following equation:

$\lambda\left( t \right)=\frac{1}{L\left( t \right)}\int_{t}^{T} \left( p\left( t' \right)-s\left( t' \right) \right)L\left( t' \right)dt'=max\frac{\left[ \begin{aligned} total net photosynthesis \\ in the rest of the year \end{aligned} \right]}{\left[ current leaf area \right]}$ (A.11)

In these equations, $s\left( t \right)$ is the value chosen along the optimal growth trajectory. In Eq. (A.11), we represent this fact explicitly by stating max symbol.

**Appendix B**


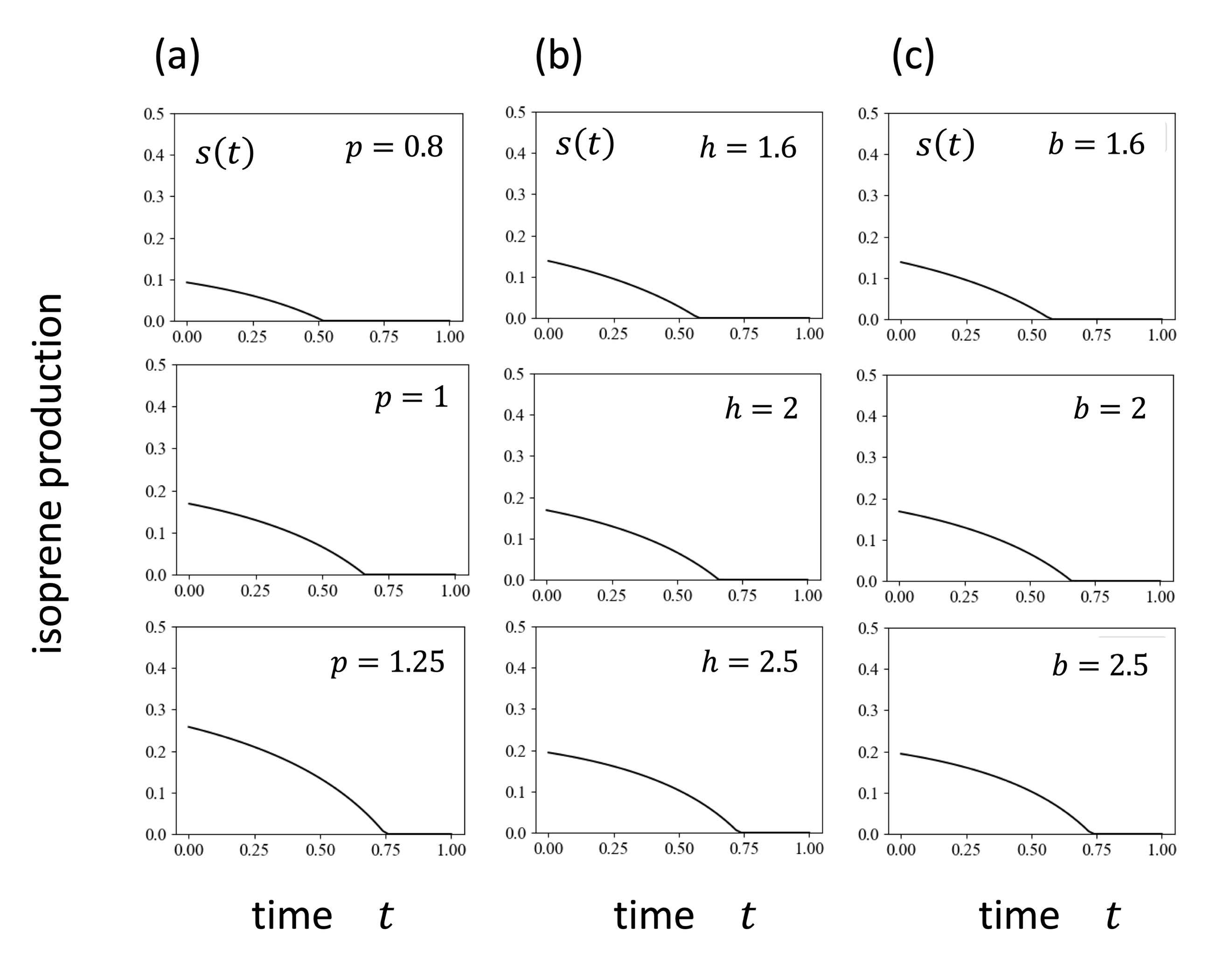
 Figs. S1 illustrates the parameter dependence of the optimal allocation to isoprene production $s\left( t \right)$ for different values of parameters when all the rates are independent of time. Figs. S1(a), 3(b), 3(c), 3(d), 3(e) illustrate the change for different values of $p$, $h$, $b$, $u$, $T$, respectively. These illustrate that the optimal isoprene production rate is larger for larger photosynthesis rate, stronger heat stress, higher effectiveness of isoprene, but is almost independent o random leaf loss rate, in this analysis. Fig. S1(e) illustrates the cases with different length of the growing season $T$. Solution given by Eqs. (4) and (5) depends on $T-t$, length of time until the end of the season.


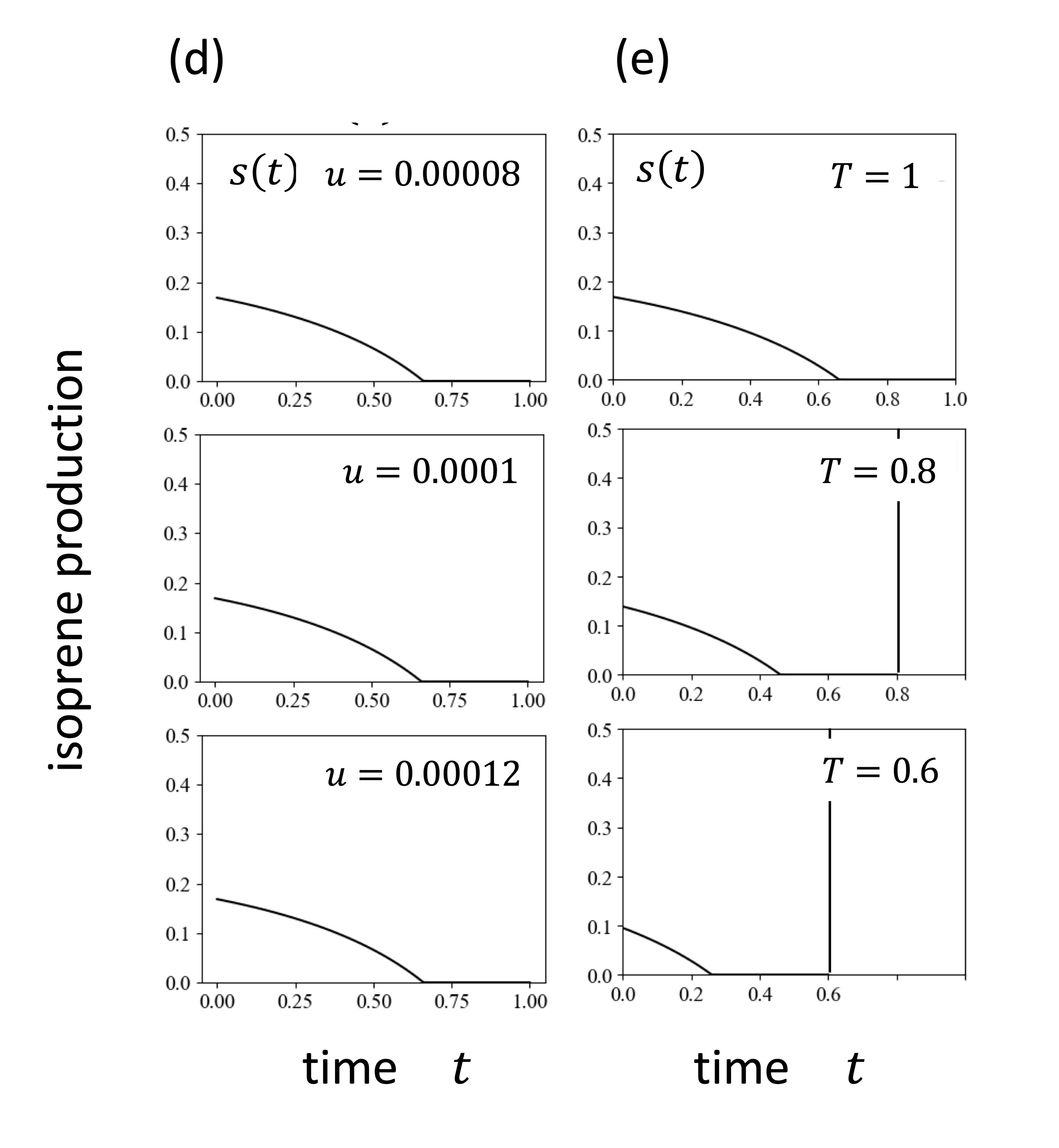


**Fig. S1** Parameter dependence of optimal schedule of isoprene production when all the rates are constant. Three parts (top, middle, bottom) show the results when the following parameters are modified one by one: (a) photosynthetic rate $p$, (b) heat stress $h$, (c) effectiveness of BVOC $b$, (c) random mortality $u$, and (d) length of the season $T$. Unless specified otherwise, parameters are as follows: $p=1$, $h=2$, $b=2$, $u=0.0001$, $T=1$. The optimal production of BVOC increases with a stronger photosynthesis (large $p$), stronger heat stress (large $h$), but was almost independent of random mortality ($u$). A larger $T$ shifts the whole schedule later. Because $s_{max}$ was large, the solutions did not depend on $s_{max}$.

***B.1 Elasticity***

To indicate parameter sensitivity, the elasticity, defined as follows, is often adopted:

$e_{y,x}=\frac{\partial logy}{\partial logx}=\frac{x}{y}\cdot\frac{\partial y}{\partial x}$ (B.1)

which is called "the elasticity of $y$ with respect to $x$" or "$x$*-*elasticity of y", indicating the change in $y$ caused by a small increase in $x$, which is adopted widely in economics and population ecology [36] [37] [38]. The value of elasticity is independent of the unit of the quantities. For example, it remains the same irrespective of meter, cm, or mm is adopted in measuring $y$ or $x$.

We examined the enhanced magnitude of $y=s\left( 0 \right)$ to depend on the parameters in the model. The values of elasticity vary depending on the standard parameters. It was anticipated that there would be a consistent trend regarding the sign and magnitude of these values. Therefore, by varying the standard parameters over a wide range and retaining only those for which all elasticities could be calculated. 74 data points were obtained. The range of elasticities for parameters included in the model is represented by box plots in Fig. S2. For parameters such as $p$, $h$, $b$, $T$, elasticities were positive and of the order of 1, while the elasticity for $u$ was negative and was considerably smaller in magnitude. From this, we confirmed that the optimal isoprene production tends to increase with a higher photosynthesis rate, increased heat stress, improved efficiency of the chemical, and a longer growing season.

**
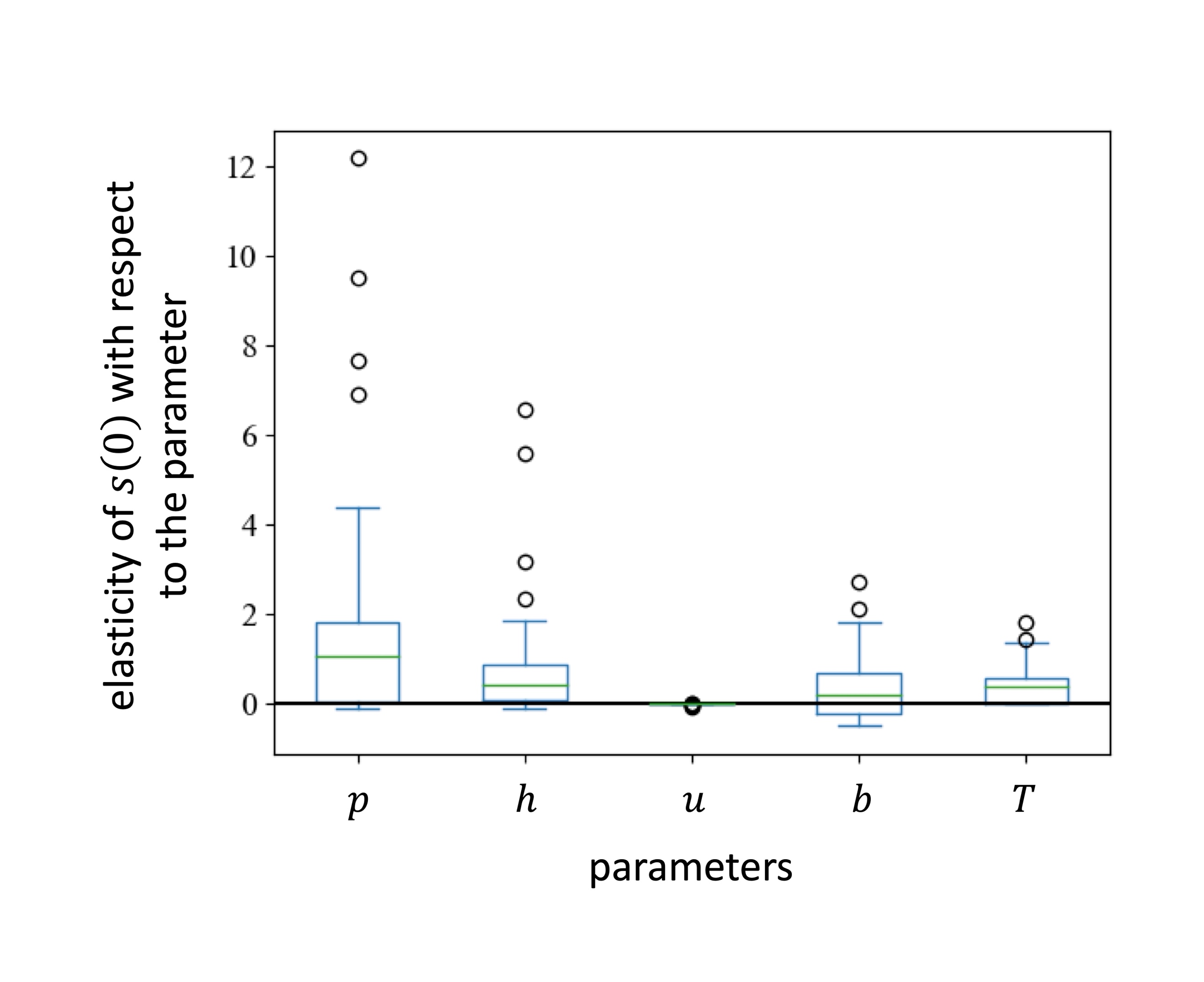
**

**Fig. S2** Box plots of elasticities of parameters in the model, measuring the effects on the maximum rate of isoprene production $s\left( 0 \right)$. We changed parameters within $0<p<2$,$0<h<4$, $0<b<3$, and $0<u<0.01$. We obtained 74 standard parameter sets for which all the elasticities were calculated. The results of elasticities are shown in box plots.

**Appendix C**

Fig. S3 indicates that, if the total heat risk $H_{total}= \int_{0}^{T} h\left( t \right)dt$ was controlled, the three cases differed in shape constant $b$ but $a$ was adjusted to keep $H_{total}$ equal. The magnitude of the difference of the peak dates between the isoprene production and the heat stress did not increase much with $b$. Figs. 4 and S3 combined suggest that the magnitude of the peak shift was more strongly controlled by total heat risk $H_{total}$ than by shape constant $b$.


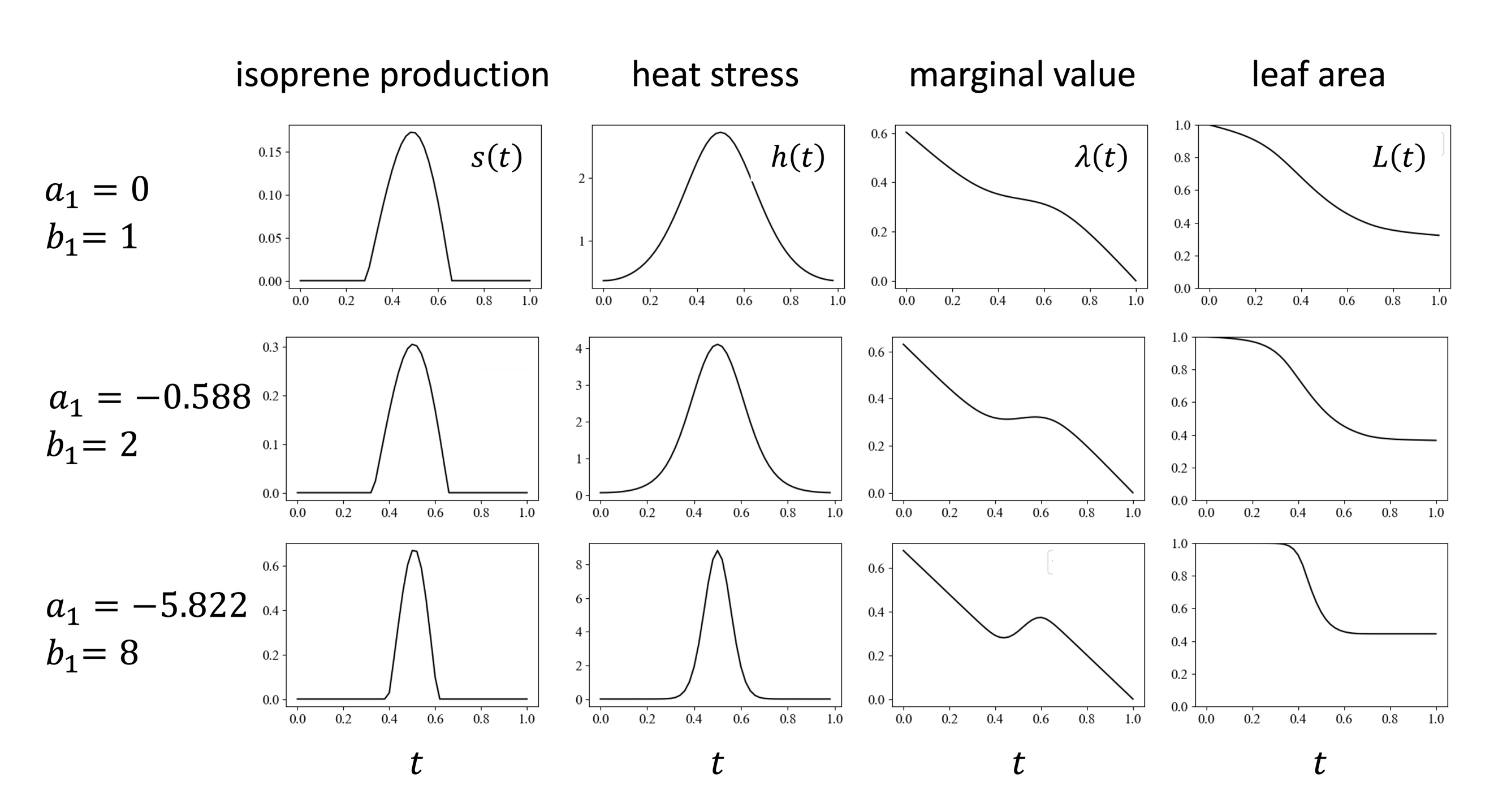


**Fig. S3** Optimal schedule of isoprene production when heat stress has a peak in the middle of the season. Three cases differ in shape factor $b_{1}$ ($b_{1}=1, 2$, and 8, for top, middle and bottom parts), and the curve of $h\left( t \right)$ became more peaky as $b_{1}$ increased. We adjusted $a_{1}$ to make $H_{total}$ the same between them ($a_{1}=1$, -0.555, -5.822). The magnitude of post-risk enhancement of marginal value of leaf area did not change much and the shift in the peak date of isoprene production did not increase. Other parameters are the same as those for Fig. 5.
